# Parent ratings of executive function in young preschool children with symptoms of attention-deficit/-hyperactivity disorder

**DOI:** 10.1186/s12993-015-0060-1

**Published:** 2015-04-15

**Authors:** Annette Holth Skogan, Pål Zeiner, Jens Egeland, Anne-Grethe Urnes, Ted Reichborn-Kjennerud, Heidi Aase

**Affiliations:** Division of Mental Health and Addiction, Oslo University Hospital, Pb 4959, Nydalen, 0424 Oslo Norway; Institute of Psychology, University of Oslo, Pb 1094, Blindern, 0317 Oslo Norway; Vestfold Hospital Trust, Pb 2267, 3103 Tønsberg, Norway; Regional Centre for Child and Adolescent Mental Health, Eastern and Southern Norway (RBUP), Pb 4623, Nydalen, 0405 Oslo Norway; Division of Mental Health, Norwegian Institute of Public Health, Pb 4404, Nydalen, 0403 Oslo Norway; Institute of Clinical Medicine, University of Oslo, Pb 1094, Blindern, 0317 Oslo Norway

**Keywords:** ADHD, Executive function, Preschool, BRIEF-P, Inhibition, Working memory

## Abstract

**Background:**

Recent research has demonstrated that deficits in basic, self-regulatory processes, or executive function (EF), may be related to symptoms of attention-deficit/hyperactivity disorder (ADHD) already during the preschool period. As the majority of studies investigating these relations in young children have been based primarily on clinically administered tests, it is not clear how early symptoms of ADHD may be related to observations of EF in an everyday context. The preschool version of the Behavior Rating Inventory of Executive Function (BRIEF-P) was developed to provide information about EF through observable, behavioral manifestations of self-regulation, and is the most commonly used rating scale for EF assessment in children.

**Methods:**

Relations between symptoms of ADHD reported in the Preschool Age Psychiatric Assessment interview (PAPA), and EF as measured by the BRIEF-P (parent form), were investigated in a large, nonreferred sample of preschool children (37–47 months, n = 1134) recruited from the Norwegian Mother and Child Cohort Study (MoBa) at the Norwegian Institute of Public Health. The inventory’s discriminative ability was examined in a subsample consisting of children who met the diagnostic criteria for either ADHD, oppositional defiant disorder (ODD) or anxiety disorder, and typically developing controls (n = 308). The four groups were also compared with regard to patterns of EF difficulties reported in the BRIEF-P.

**Results:**

Of the five BRIEF-P subscales, Inhibit and Working Memory were the two most closely related to ADHD symptoms, together explaining 38.5% of the variance in PAPA symptom ratings. Based on their scores on the Inhibit and Working Memory subscales (combined), 86.4% of the children in the ADHD and TD groups were correctly classified. ADHD symptoms were associated with more severe difficulties across EF domains, and a different EF profile in comparison to children with other symptoms (anxiety, ODD) and to typically developing controls.

**Conclusions:**

Early symptoms of ADHD were linked to parent-reported difficulties primarily within inhibition and working memory, suggesting that deficiencies within these two EF domains characterize early forms of ADHD. Our findings support the clinical utility of the BRIEF-P as a measure of EF in young preschool children with symptoms of ADHD.

## Introduction

Deficiencies in basic self-regulatory processes- collectively referred to as executive function (EF), are frequent among children with attention-deficit/hyperactivity disorder (ADHD). The association is well-documented in school-aged samples [1], and during recent years, evidence from studies of preschool children has indicated that symptoms of ADHD may be linked to deviances in EF already during the first preschool years [2,3]. The majority of research addressing the association between EF and early symptoms of ADHD relies on performance-based measures- usually clinically administered tests- which provide information about specific cognitive abilities involved in self-regulation, such as working memory and inhibition [4]. Several factors, however, are likely to affect the way these capacities become apparent in an everyday setting, and information from performance-based measures of EF needs to be complemented with observations of self-regulation in natural, everyday contexts [5-8].

The Behavior Rating Inventory of Executive Function (BRIEF) [9] was specifically designed to assess EF via observable, behavioral manifestations of the specific cognitive processes involved in self-regulation. The inventory has become the most commonly used rating scale for assessment of EF in children [8] and has proven to be sensitive to EF impairment in several early-debuting developmental disorders such as ADHD, oppositional defiant disorder (ODD) and autism spectrum disorders [10-12]. Although less consistent, findings from these and other studies of EF in children with various difficulties have provided support for the inventory’s discriminative ability [10,13,14]. The preschool version (BRIEF-P) is a modification of the original inventory, for use with children aged 2 through 5 years [15]. It is less studied than the school-age version, but evidence so far supports its clinical utility in the preschool population [12,16,17].

Given the limited knowledge about relations between everyday executive functioning and early signs of ADHD at the point in development when fundamental EF processes are thought to emerge, the present study’s main objective was to investigate associations between EF as measured by the BRIEF-P, and ADHD symptoms in young preschool children. Relations between ADHD symptoms and behavioral ratings of EF were studied in a large, nonreferred sample of 3-year -old preschoolers. We first examined the amount of variance in ADHD symptoms predicted by each of the five BRIEF-P subscales. The inventory’s ability to discriminate between children who met diagnostic criteria for ADHD and typically developing controls was then assessed in a subsample. In a last set of analyses, we compared patterns of EF difficulties across four groups: three with children who met diagnostic criteria for ADHD, ODD and anxiety respectively, and one consisting of typically developing children.

### EF and ADHD in preschool age children

Executive functions are commonly referred to as higher-order cognitive abilities necessary for goal-directed behavior [18]. The preschool period (ages 3–6 years) is considered to be particularly important in the development of these skills, which are crucial to self-regulation. According to studies of normative EF development, elementary forms of its two core components- inhibition and working memory (WM)- are established during the first preschool years [19,20]. They are in turn thought to underlie a wide range of more complex regulatory processes that show a more protracted developmental course; such as mental flexibility, planning and organizing [21,22]. It has been demonstrated that basic EF components may be differentiated and assessed in preschool children by the use of developmentally appropriate tasks [4,23]. The correspondence between performance-based measures and behavior ratings of EF tends to be poor [6,8], but basic executive capacities measured by neuropsychological tests- have been shown to support broader control processes enabling self-regulation across both cognition and emotion in preschool children [24].

Preschool children with symptoms of ADHD resemble their school-aged counterparts with regard to EF, with deficits primarily in inhibition and, to a lesser degree, in WM [2]. However, associations between EF and symptoms of ADHD may vary with age, even within the preschool period. The specificity of EF deficits in ADHD has been questioned, as difficulties within core EF domains have been described in several other clinical groups [25]. Among these are two of the other most common psychiatric disorders among preschoolers: ODD and anxiety. ODD is a frequent, co-occurring disorder in preschool children with ADHD [26], and is often associated with poorer EF. Early symptoms of ODD have been linked to difficulties in inhibition and emotional regulation [3,27]. ODD-related deficits in WM have been described in preschool children [28]; recent research has indicated, however, that the relation between ODD and poor performance in tests of WM may be accounted for by co-occurring symptoms of ADHD [29,30].

EF difficulties have also been described in children with internalizing problems, such as anxiety. Links between anxiety and reduced mental flexibility (shifting) have been demonstrated both in preschool- and school-aged samples [31,32]. According to process efficiency theory, anxiety problems are likely to put a strain on information processing and storage in WM [33,34]. Research addressing WM in anxious children has, however, reported mixed findings; with an association described by some [31,35,36] but not others [32,37].

Although most frequently described in children with ODD, recent research suggests that emotional dysregulation contributes to the symptomatology of all three of the abovementioned disorders [38-40]. In a recent study conducted by our research group, emotional lability (EL) as measured by the BRIEF-P subscale Emotional Control was shown to be associated with early symptoms of ADHD, ODD and anxiety [41].

To the best of our knowledge, only one previous study has investigated parent ratings of children with elevated levels of ADHD symptoms using the complete BRIEF-P. Comparing parents’ ratings of EF in 25 children aged 3–5 years who were diagnosed with ADHD, with 25 normally developing, matched controls, Mahone and Hoffman found that children with ADHD were rated as more impaired than children without ADHD on all five BRIEF-P subscales. The strongest effect was found for the Working Memory subscale [42]. However, possible age- and disorder specific links between behavioral dysregulation and ADHD symptoms may however have gone undetected in this study. Conclusions were based on pooled data from 3-5-year old children, and BRIEF-P ratings of children with ADHD were only compared with normally developing controls. A small sample size, and an exclusively categorical approach to ADHD may also have served to obscure associations between variables of interest.

### Aims and hypotheses

In the present study, our first aim was to investigate possible links between early symptoms of ADHD and parent ratings of EF on the BRIEF-P’s five clinical subscales. In our young preschool sample, we expected symptoms of ADHD to be related primarily to the two basic EF domains inhibition and WM. Secondly, we investigated our hypothesis that the BRIEF-P Inhibit and Working Memory subscales would discriminate accurately between children in our sample who met the diagnostic criteria for ADHD, and typically developing controls. Thirdly, we aimed to extend previous research on EF in young children with ADHD by examining and comparing their BRIEF-P profile in relation to profiles of children with symptoms of ODD, anxiety, and typically developing controls. All three clinical groups were expected to exhibit higher overall BRIEF-P problem scores than did typically developing children. We expected that externalizing problems (ADHD, ODD) would be associated with higher problem ratings primarily on the Inhibit scale relative to typically developing children, but to differ with regard to scores on the Working Memory scale. We further hypothesized that anxious children would present with fewer inhibitory problems than the other two clinical groups, but would show poorer mental flexibility as reflected in the BRIEF-P Shift subscale. As empirical findings with regard to WM in this clinical group have been inconsistent, the question with regard to anxiety-related deficiencies in WM was considered to be open.

## Methods

### Participants

The present study used data from a longitudinal prospective study of ADHD (The ADHD study) with participants recruited from The Norwegian Mother and Child Cohort Study (MoBa), a population based birth cohort study managed by the Norwegian Institute of Public Health [43]. When each child reached 36 months, MoBa participants received a questionnaire including 11 questions regarding hyperactivity, impulsivity and attention problems; six from the Child Behavior Checklist [44] and five from the DSM-IV diagnostic criteria for ADHD [45]. In order to oversample children with relevant symptoms, about 80% of children invited to the clinical assessment were drawn from those who scored at or above the 90th percentile on these questions, and/or whose parents reported hyperactivity as a health problem (later referred to as screen positive). A total of 2 798 children were invited from the MoBa during the period from August 2007 to January 2011 based on these criteria. Of these, 1 048 children (37.5%) participated in the clinical assessments. An additional 654 children randomly selected from the MoBa cohort within the same time frame were invited, of whom 147 (22.5%) participated. The total number of children who were clinically assessed was therefore 1 195. Children taking part in the ADHD study had older mothers with slightly higher educational levels and fewer children than those who declined to participate [Biele, personal communication, October 2014]. Inclusion in the present study required a BRIEF-P parent form with fewer than 12 missing responses in total, and fewer than two missing responses within any single subscale. For those included, missing scores were replaced with item score 1 (n = 110 children) consistent with scoring instructions [15]. Our total sample thus consisted of 1 134 children (544 girls, 590 boys), aged 37 to 47 months. Investigation of correlations and analyses exploring relations between behavioral symptoms of ADHD and BRIEF-P ratings across symptom severity were conducted in the entire sample. The discriminative ability of the BRIEF-P and EF profiles were investigated in a subsample (n = 308), as described below.

None of the participating children had been or was receiving psychopharmacological treatment for ADHD at the time of assessment. Parents gave informed consent to the research and to the publication of the results. The MoBa and the present substudy were approved by the Norwegian Regional Committee of Ethics in Medical Research and The Norwegian Data Inspectorate.

### Procedure and measures

Upon accepting the invitation to participate in the ADHD prospective study, parents were requested to fill out the BRIEF-P and return it by the time of assessment. This request was made approximately four weeks prior to the one- day clinical assessment, which included a structured clinical interview with one of the parents (see below), a short medical screening, and a neuropsychological examination. All information from the clinical assessments and the parent interview was reviewed by senior specialists, who assessed symptom quality and severity, and the validity of test results.

### BRIEF-P

The BRIEF-P consists of 63 items that describe children’s everyday executive function behaviors. Parents respond regarding whether their child exhibits problems with specific behaviors; Never (1), Sometimes (2) or Often (3). Thus, higher scores are associated with poorer executive functioning. Ratings are summed across items within each of the five theoretically and empirically derived subscales: *Inhibit* (16 items, range 16–48), *Shift* (10 items, range 10–30), *Emotional Control* (10 items, range 10–30), *Working Memory* (17 items, range 17–51) and *Plan/Organize* (10 items, range 10–30). The subscales are then summarized into three broad indexes: Inhibitory Self-Control (ISC; Inhibition and Emotional Control), Flexibility (FI; Shift and Emotional Control), and Emergent Metacognition (EMI; WM and Plan/Organize). Data collection in the prospective study commenced in 2007, using an existing Norwegian translation developed for research purposes [46]. A new BRIEF-P translation, closer to Gioia et al.’s original version, became available for research purposes in 2009, and was implemented in the second half of the data collection process (from 2009 to 2011). Because a larger proportion of recruited children were screened as positive in the latter half of the data collection process, BRIEF-P scores from the two translations were compared at the item level via confirmatory factor analysis. A comparison of four different models of inter-item relations, which allowed same and/or different factor means and factor loadings for the two BRIEF-P translations, showed that the best solution was the one assuming the same loadings and different means. On the basis of these results, data from the two translations were combined in the present study.

### Psychiatric symptoms

Psychiatric symptoms were assessed using an adapted version of the Preschool Age Psychiatric Assessment interview (PAPA) [47]. The PAPA interview was developed for use with children aged 2–6 and provides information about the scale and frequency of criteria according to diagnoses in the DSM- IV-TR, including information on impairment. Impairment was considered to be present if parents reported the child to be impaired by psychiatric symptoms in one or more areas of functioning. Interrater reliabilities (intraclass consistency) of the number of DSM-IV symptoms assessed by PAPA in the present study were .98 for total number of both ADHD and ODD symptoms, and .86 for anxiety.

For the categorical analyses, participants were selected to one of four groups based on information from the PAPA interview: ADHD (n = 104), ODD (n = 39), anxiety (ANX; n = 48) or typically developing controls (TD; n = 117). Children were assigned to the ADHD group if they were reported to have at least six of nine DSM-IV-TR criteria of inattentive subtype and/or hyperactive/impulsive subtype ADHD. The ODD group comprised children with at least four of eight symptoms of ODD according to DSM-IV criteria. Inclusion in either the ADHD or the ODD group required impairment, and symptom duration of three months or more. Children with co-occurring ODD/ADHD were excluded from the ADHD and the ODD groups. Children exhibiting symptoms of one or more of the most frequent DSM-IV anxiety subtypes (i.e., specific phobia, social anxiety, separation anxiety and generalized anxiety) were assigned to the ANX group if their anxiety symptoms were inappropriate and excessive, and caused impairment. The TD group consisted of children randomly drawn from the MoBa cohort, who did not meet criteria for any psychiatric condition.

### Covariates

Child gender and mother’s educational status were included as covariates, as these variables are likely to be related to the severity and type of ADHD symptoms [48-50]. ADHD is typically associated with lower estimates of general intellectual ability, as measured by a test of intellectual function (IQ) [51,52] when compared with normally developing controls. Much remains to be understood, however, about how IQ may affect the relationship between ADHD and EF [53,54]. In the present study, we chose to include IQ as a predictor in the dimensional analyses, where its contribution to explained variance in ADHD symptoms could be estimated. An abbreviated IQ score was derived from the child’s score on the Vocabulary and Object Matrices subtests of the Stanford-Binet Intelligence Scales (5th edition) [55]. The correlation between the abbreviated IQ score and full scale IQ score as estimated by the Stanford-Binet scales is reported to be .81 in the standardization sample’s youngest age group (2–5 years) [56]. Mother’s educational status was measured by years of completed education at the time of enrollment into MoBa (i.e., at week 17 of pregnancy). Child age was not included as a possible confounder, because of the narrow age range and absence of significant associations between age and ADHD symptom load.

### Statistical analyses

Data analyses were conducted using PASW 21.0, except for the comparison of the two BRIEF-P translations, which was performed in Mplus 7.11. Descriptive data were computed for child characteristics, maternal education, symptom ratings from the PAPA interview, and BRIEF-P scores. Pearson’s correlation coefficient was used to study relations between parent-reported symptoms of ADHD from the PAPA, maternal education, general intellectual ability, and BRIEF-P ratings. Missing analyses revealed no significant differences between children included in the data set, and those excluded because of an incomplete BRIEF-P form (n = 38) with regard to any of these variables [57].

Dimensional analyses were performed in the entire sample in order to assess the amount of variance in ADHD symptom load (total number of ADHD symptoms reported in PAPA) explained by the BRIEF-P. The first set of linear regression analyses (univariate) assessed the amount of variance in ADHD symptom load, explained by each of the five BRIEF-P subscales. Gender, maternal education, and IQ were entered as predictors in step one, and each subscale in five separate analyses in step two. Addressing our hypothesis of the two subscales Inhibit and Working Memory as primary contributors to variance in ADHD symptom load, the contribution to explained variance in ADHD symptoms by the Inhibit and Working Memory subscales relative to the remaining three scales was further explored by use of hierarchical regression analysis.

The categorical analyses were conducted in a subsample. BRIEF-P subscale scores and indexes in the four groups were first compared in a multiple analysis of variance (MANOVA). The Inhibit and Working Memory subscales’ ability to predict group adherence correctly among children with ADHD and typically developing controls was then estimated by use of a discriminant function analysis. The four groups (ADHD, ODD, ANX and TD) then served as the between-group variable, and each of the five BRIEF-P subscale scores as within-subject variables in a profile analysis which allowed for the comparison of subscale profiles among the four groups, asking three questions: 1) Do the profiles show the same level of scores (severity of EF difficulties equal across groups)? 2) Do any of the profiles exhibit flatness across subscales (severity of EF difficulties equal across EF domains)? and 3) Are the scoring profiles from the four groups parallel (elevations on the same or different subscales)?

## Results

Means and standard deviations for age, child IQ, maternal education, and number of parent-reported psychiatric symptoms in the total sample are displayed in Table 1. Correlations between maternal education, IQ, parent-reported symptoms of ADHD, and BRIEF-P subscales for the total sample are reported in Table 2. Pearson correlations between BRIEF-P and ADHD symptom load were significant for all subscales and indexes. ADHD symptoms were strongly correlated with the Inhibit (r = .62) and Working Memory scales (r = .56). Medium to small correlation coefficients signified a weaker relationship between ADHD symptom load and the three remaining subscales, with coefficients ranging from .27 (Shift) to .49 (Plan/Organize).

**Table 1:** **Means and standard deviations for age, IQ, maternal education and parent-reported symptoms from PAPA (N = 1134)**

	**M**	**SD**	**Range**
Age (Months)	41.8	1.3	37-47
*IQ*	101.8	9.2	70-130
Maternal education (Years)	15.3	2.3	9-18
*PAPA symptom load*			
ADHD	4.0	3.9	0-18
ODD	1.5	1.5	0-8
Anxiety	0.8	1.2	0-8

Note: IQ = Abbreviated IQ from Stanford Binet 5th Edition. PAPA = Preschool Age Psychiatric Assessment interview. PAPA Symptom load = Number of symptoms reported in the clinical parent interview.

**Table 2:** **Correlations Between ADHD Symptoms, Maternal Education, IQ and BRIEF-P Subscales and Indexes (N = 1134)**

	**BRIEF-P**							
	**Inhibit**	**Shift**	**Emotional control**	**Working memory**	**Plan/Org**	**ISCI**	**FI**	**EMI**
ADHD symptoms	.62***	.27***	.35***	.56***	.49***	.58***	.36***	.57***
Maternal education (years)	-.13***	-.10**	-.07*	-.11**	-.10	-.11***	-.09**	-.11***
IQ	-.09*	-.10**	-.06*	-.10***	-.08*	-.09*	-.09*	-.10*
BRIEF-P								
Inhibit								
Shift	.45***							
Emotional Control	.58***	.52***						
Working Memory	.75***	.47***	.49***					
Plan/ Organize	.67***	.41***	.51***	.78***				

Note. *p < .05; **p < .01, ***p < .001.

### Dimensional analyses

Results from the univariate and multiple hierarchical regression analyses performed in the full sample are presented in Table 3. Gender, IQ and maternal education, entered together in step 1, explained 3.1% of the variance in ADHD symptom load; *F* change (3, 1107) = 11.88, p < .001). Each of the five BRIEF-P subscales contributed significantly to the model in the univariate analyses (*Δ* R^2^s ranging from .06 to .37). The relative contribution of the subscales to variance in the dependent variable was examined in the multiple analyses: Entered simultaneously in step two, the five subscales explained 38.9% of the variance in ADHD symptom load; *F* change (5, 1102) = 147.68, p < .001. Three subscales contributed significantly to the model as a whole; Inhibit, Working Memory and Shift. A further investigation of the first two predictors’ contribution to explained variance in ADHD symptoms revealed that of the 38.9% explained by all five subscales, Inhibit and Working Memory accounted for 38.5%. The addition of Shift, Emotional Control and Plan/Organize scales to the model led to a marginal change in this estimate (*Δ R*^*2*^ = .004; p = .053).

**Table 3:** **Summary of Linear Regression Analyses for BRIEF-P Predicting number of ADHD symptoms (N = 1134)**

	**Univariate analyses**					**Multiple analyses**			
	**B**	***SE B***	**β**	**p**	Δ ***R*** ^***2***^	**B**	***SE B***	**β**	
Gender	-.56	0.23	-.07	.014	.005				
Maternal education	-.25	0.05	-.15	.000	.022				
IQ	-.04	0.01	-.10	.001	.010				
BRIEF-P subscales									
Inhibit	.40	0.02	.62	<.001	.365	.31	0.03	-.47	<.001
Shift	.32	0.04	.25	<.001	.059	-.08	0.04	-.06	.032
Emotional Control	.35	0.03	.35	<.001	.120	-.02	0.03	-.02	.522
Working Memory	.39	0.02	.55	<.001	.296	.14	0.03	.20	<.001
Plan/Organize	.54	0.03	.48	<.001	.226	.05	0.04	.05	.222

*Note.* In the multiple analyses, gender, maternal education and IQ were entered together in step 1. Each of the five BRIEF-P subscales were entered in step 2 in five separate hierarchical analyses. Dependent variable: Number of ADHD symptoms.

### Categorical analyses

Means and standard deviations for BRIEF-P subscales and indexes for the four comparison groups are shown in Table 4, together with results from the multivariate analysis of variance (MANOVA). Children in the four groups did not differ significantly with respect to age, IQ or maternal education. There was a preponderance of boys in the ADHD and TD groups, while gender distribution was close to equal in the ODD and Anxiety groups.

**Table 4:** **Means and Standard Deviations for BRIEF-P Subscores and Indexes and Results of the Multivariate Analysis of Variance (MANOVA) (n = 308)**

**Group**						
**(Girls/boys)**	**ADHD**	**ODD**	**ANX**	**TD**		
	**n = 104**	**n = 39**	**n = 48**	**n = 117**		
	**(38/66)**	**(18/21)**	**(21/27)**	**(52/65)**		
	**M (SD)**	**M (SD)**	**M (SD)**	**M (SD)**	**F (9,906)**	**η**_**p**_^**2**^
BRIEF-P raw scores						
Inhibition	30.8 (6.1)	26.6 (5.3)	23.8 (4.8)	20.2 (4.1)	81.9	.45
Shift	14.1 (3.2)	14.0 (3.0)	15.5 (3.6)	11.9 (2.5)	19.5	.16
Emotional Control	17.2 (4.2)	18.4 (4.2)	16.8 (3.8)	13.1 (3.1)	32.6	.24
Working Memory	28.6 (6.3)	24.6 (4.2)	24.7 (5.8)	20.7 (4.2)	41.7	.29
Plan/Organize	17.5 (3.8)	16.3 (2.8)	15.2 (3.4)	13.0 (2.7)	37.0	.27
BRIEF-P indexes						
*ISCI*	48.0 (9.1)	45.0 (8.7)	40.6 (7.3)	33.2 (6.5)	68.6	.40
*FI*	31.3 (6.3)	32.4 (6.2)	32.3 (6.6)	25.0 (4.8)	33.6	.25
*EMI*	46.2 (9.5)	40.9 (6.1)	39.9 (8.7)	33.7 (6.5)	45.0	.31

Note. All results were significant at level p < .001. ISCI = Inhibitory Self-control Index, FI = Flexibility Index, EMI = Emergent Metacognition Index.

To assess the accuracy of the BRIEF-P subscales Inhibit and Working Memory in predicting group membership (ADHD or TD), we conducted discriminant function analyses with the two subscales entered simultaneously as predictors. Of the 221 children in the ADHD and TD groups, 86.4% (cross-validated cases) were correctly classified on the basis of their scores on these two subscales. In the ADHD group, 80.1% of the children were correctly classified; the corresponding percentage in the TD group was 91.5%. In a second discriminant analysis, with the same two subscales as predictors but with all four groups included, the percentage of correctly classified children was 55.8.

**Figure 1:**
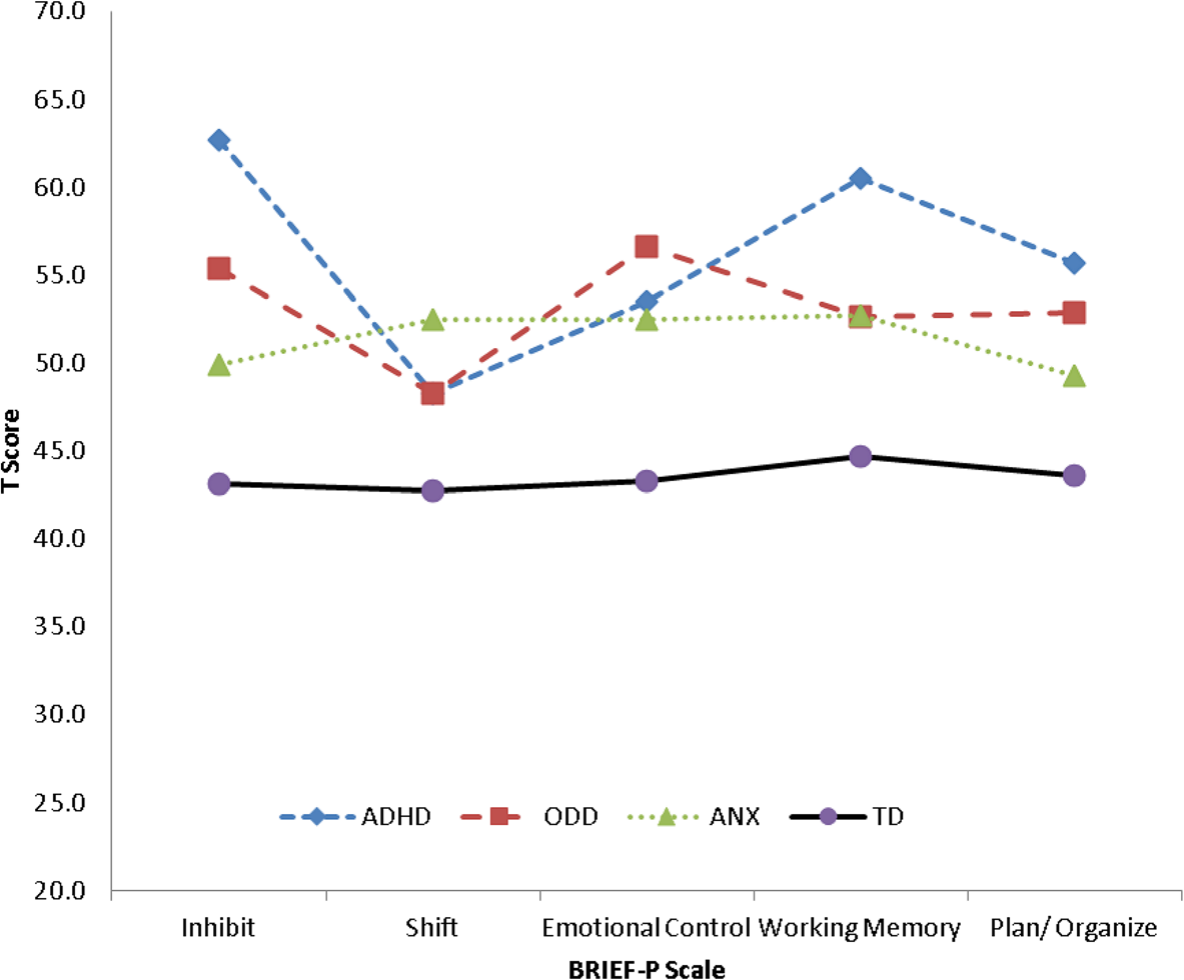
Profiles for ADHD, ODD, ANX and TD Groups Accross BRIEF-P Scales.

MANOVA results (Table 4) revealed an overall group effect for the five BRIEF-P subscales (F (15, 906) = 17.9, p < .001, Pillai’s Trace = .78; η^2^ = .23) and indexes (F (9,912) = 26.4, p < .001, Pillai’s Trace = .62; η^2^ = .21) which remained significant after controlling for gender. Group differences across BRIEF-P subscales were further explored in a profile analysis (Figure 1). Results revealed a significant difference between groups in scale scores (averaged across all subscales) (F (3,304) = 58.0, p < .001, η^2^ = .36), indicating differences in levels of parent-reported EF difficulties. A significant within-groups main effect was found, showing different scale elevations across the BRIEF-P subscales (F (3, 1042) = 23.1, p < .001, η^2^ = .07). Finally, the profiles of scale elevations varied between our four groups (F (10, 1042) = 19.0, p < .001, η^2^ = .16). Post hoc comparisons conducted with a conservative probability level (p < .01) indicated that children in the ADHD group scored significantly higher than typically developing controls (TD) in all five EF domains. This was also the case for the two other clinical groups (ODD, ANX). The ADHD group exhibited more problems on two of the five BRIEF-P subscales, Inhibit and Working Memory, than children in the ODD and ANX groups. In terms of absolute scores, anxious children presented with the highest problem scores of all four groups on the Shift subscale; but the only difference to reach significance was between the ANX group and typically developing controls.

## Discussion

Relationships between early symptoms of ADHD and behavioral ratings of executive function were investigated in a large sample of young preschool children. Symptoms of ADHD were found to be associated with parent-reported difficulties within core self-regulatory processes. The present study extends previous research by demonstrating that these relationships are identifiable from early on in the preschool period.

Consistent with our first hypothesis, the BRIEF-P Inhibit and Working Memory scales were most closely related to ADHD symptoms in our sample. With regard to inhibition, this result corresponds to a recent study of relations between disruptive behavior (ADHD, ODD) and teacher ratings of EF in 3-year old children, using the BRIEF-P [58]. It is also consistent with research based on neuropsychological test data, demonstrating a relatively robust relationship between symptoms of ADHD and inhibitory problems in preschool children [2].

Findings have so far been less consistent with regard to the role of WM in early ADHD, with only a handful of preschool studies reporting ADHD-related difficulties in WM as measured by neuropsychological tests ([29,30,59,60], but see [61,62]). The present study is one of two based on parents’ behavioral ratings of WM in preschoolers with elevated levels of ADHD symptoms: both reported evidence for a link between BRIEF-P ratings of WM and early symptoms of ADHD. Although noteworthy, this finding does not constitute a sufficient basis for conclusions with regard to a definitive role of WM in early forms of ADHD, and further research is warranted on the role of WM in the development of ADHD symptoms and impairments.

Scores on the Inhibit and Working Memory subscales were shown to discriminate well between children with symptoms of ADHD and typically developing controls in the categorical analyses; Inhibit and Working Memory scores in the ADHD group exceeded the corresponding scores in the control group by 1.5 standard deviations. Thus, the present findings support the clinical utility of the BRIEF-P in differentiating between children with clinically significant symptoms of ADHD and typically developing controls. It is noteworthy that none of the five mean subscale scores exceeded the suggested threshold for clinical significance (T > 65) for any of the three clinical groups when the original US norms were applied. Contrary to previous findings in clinical samples, this may be related to characteristics of the present sample- including possible cultural differences- or perhaps to a somewhat stronger reluctance among parents to describe certain behaviors as problematic at this early point in development [26,63]. In light of this, the overall percentage of correctly categorized children in our sample (86.4% based on the Inhibit and the Working Memory scales) was surprisingly similar to that reported in studies of school-aged children [11,64].

All five BRIEF-P subscale scores were clearly elevated for children with symptoms of ADHD, ODD or anxiety in comparison with their typically developing peers. Overall, 3-year old children with externalizing problems (ADHD, ODD) were reported to have more pronounced difficulties as measured by the BRIEF-P than children with internalizing problems (i.e., anxiety). While the TD group exhibited a relatively flat EF profile, indicating no particular problems in any domain, the three symptom groups differed in several ways, both from each other and relative to controls. Children in the ADHD group were rated as having more problems within the inhibition and WM domains than the ODD and ANX groups, and the controls. The observed pattern is consistent with what was predicted on the basis of previous research, and it suggests that difficulties within the two EF domains may be characteristic for ADHD at this early developmental stage. It is noteworthy that DSM-IV criteria for the ADHD diagnosis and formulations in some of the BRIEF-P items, particularly in the Inhibit and Working Memory scales, are different in content but similar in concept [13]. This is likely to have contributed to this result, as well as to estimates of the inventory’s predictive ability.

The ADHD and the ODD group had in common a peak on the Inhibit problem scale; however, the second peak for the ADHD group was found in the Working Memory scale, while the second peak for the ODD group was on the Emotional Control scale. The closer relationship between difficulties in emotional control and early symptoms of ODD relative to other symptoms in the present study is consistent with previous research on older children, which describes emotional dysregulation as a core feature of ODD [65,66]. Although scarce, studies of preschool children have indicated that this link may be present from early on in development [27,58]. One of these studies was based on longitudinal data, and reported that ODD in middle childhood was predicted by emotional dysregulation as measured by an emotional temperament scale at the age of 3 years [27].

We found support for the assumption that children with anxiety disorders would present with a pattern of EF difficulties different from the two other clinical groups in our sample. Anxiety symptoms were linked to relatively fewer inhibitory problems, but to the highest absolute score on the Shift scale among the three symptom groups. Although nonsignificant, the difference relative to the ADHD and ODD groups on the Shift scale may represent a first indication of an emerging, specific link between anxiety and reduced mental flexibility. Anxious children did not differ significantly from the two other clinical groups in the Working Memory scale, contrary to what could have been predicted on a theoretical basis [34]. To the best of our knowledge, only one previous study has investigated WM in children with anxiety problems using behavioral ratings to assess WM; Sørensen and colleagues [32] found no significant association between parent ratings (BRIEF) of WM and anxiety in a sample of primary school children. Taken together, these findings suggest a lack of an association between anxiety and parent ratings of WM difficulties that may not be specific to preschool children. At the same time, it is important to note that parent ratings of early WM are likely to capture other aspects of WM than do performance-based measures. Clinically administered tests are developed to assess the specific cognitive processes implicated in WM, whereas behavior ratings reflect how these processes play out in real-world, natural settings [8]. Thus, our results does not preclude the existence of an association between anxiety and WM as measured by clinically administered tests in this age group. The above findings indicate that different psychiatric symptoms, or symptom clusters, may be associated with different patterns of EF difficulties as measured by the BRIEF-P from early on in the preschool period. However, considerable individual differences, together with the observed overlap between the three clinical groups, warn against using the inventory as a diagnostic tool. This has previously been emphasized by the BRIEF authors [67].

### Strengths and limitations

The study’s most important strengths include a large sample with a fairly equal gender representation, and a narrow age range that is rarely studied with regard to ADHD and to the BRIEF-P. Psychiatric symptoms were assessed using a well-validated psychiatric interview for preschoolers, and ADHD was characterized both categorically and dimensionally.

Among limitations, it must be noted that both the MoBa cohort and the longitudinal study had relatively low participation rates (approximately 35- 40%). This has led to an underrepresentation of children from high-risk families (low socioeconomic status, young mothers, single parent families), smoking during pregnancy, and possibly children with the most severe behavioral and cognitive problems [68,69]. In the present study, this selection may have affected estimates of relationships between variables of interest through restricted variance. Investigations of exposure-outcome measures in the MoBa cohort versus the population, together with findings from a similar Danish birth cohort, suggest that the effect of this bias is limited and not likely to represent a validity problem [70,71]. It should also be noted that because of oversampling of children with elevated levels of ADHD-like symptoms, the present findings may not be directly applicable to the preschool population in general. They are, however, considered to be particularly relevant in clinical settings, addressing relations between EF and ADHD-like symptoms in a group of children with behavioral problems sufficient to raise concern in their parents.

The exclusion of children with co-occurring ADHD and ODD offered the opportunity to study possibly diagnosis-specific patterns of EF deficiencies. This precluded investigations of possible additive or interaction effects associated with this frequently occurring comorbidity. The narrow age range also limited the generalization of findings to the youngest preschoolers. As only parent ratings were included here, findings may not apply to the use of the inventory by teachers. Finally, estimates of common variance reported in the present study must be interpreted bearing in mind that assessment of psychiatric symptoms and executive behavior were both based on parent reports.

## Conclusions

The current study is one of very few to investigate relations between ADHD symptoms and behavioral ratings of EF at age 3, when basic EF skills are thought to emerge. Our findings support the use of the BRIEF-P in the identification and description of EF difficulties in young preschool children with symptoms of ADHD. The comparison of ratings within the BRIEF-P’s five clinical scales proved useful in distinguishing ADHD from the two other most commonly occurring disorders in the preschool population- ODD and Anxiety- , and from typically developing controls. ADHD-related difficulties were identified primarily in inhibition and WM at age 3 years, suggesting that deficiencies within these two EF domains contribute to the development of ADHD. These relations should be further addressed in follow-up studies of children first assessed as young preschoolers. Developed to describe patterns of behavior associated with different aspects of EF, the BRIEF-P may contribute important, ecologically valid information about early, self-regulatory capacities. Behavior ratings of EF in everyday situations are likely to tap into aspects of EF other than those measured by clinically administered tests, thus constituting an important supplemental source of information in the assessment of EF across childhood. Identification of deviancies within specific areas of everyday EF at an early point in development may aid the development of targeted interventions for use with young children.

## Abbrevations

BRIEF-P: The Behavior Rating Inventory of Executive Function
EF: Executive function
ODD: Oppositional defiant disorder
WM: Working memory
